# Hepatoprotective Effects of Glycyrrhetinic Acid on Lithocholic Acid-Induced Cholestatic Liver Injury Through Choleretic and Anti-Inflammatory Mechanisms

**DOI:** 10.3389/fphar.2022.881231

**Published:** 2022-05-31

**Authors:** Qian Wang, Guo-Chao Song, Feng-Yi Weng, Bin Zou, Jing-Yi Jin, Dong-Ming Yan, Bo Tan, Jing Zhao, Yue Li, Fu-Rong Qiu

**Affiliations:** Laboratory of Clinical Pharmacokinetics, Shuguang Hospital Affiliated to Shanghai University of Traditional Chinese Medicine, Shanghai, China

**Keywords:** cholestatic liver injury, TLRs/NF-κB signaling pathway, inflammatory cytokines and chemokines, FXR, glycyrrhetinic acid

## Abstract

Cholestasis is a clinical syndrome triggered by the accumulation and aggregation of bile acids by subsequent inflammatory responses. The present study investigated the protective effect of glycyrrhetinic acid (GA) on the cholestatic liver injury induced by lithocholic acid (LCA) from both anti-inflammatory and choleretic mechanistic standpoints. Male C57BL/6 mice were treated with LCA twice daily for 4 days to induce intrahepatic cholestasis. GA (50 mg/kg) and pregnenolone 16α-carbonitrile (PCN, 45 mg/kg) were intraperitoneally injected 3 days before and throughout the administration of LCA, respectively. Plasma biochemical indexes were determined by assay kits, and hepatic bile acids were quantified by LC-MS/MS. Hematoxylin and eosin staining of liver sections was performed for pathological examination. Protein expression of the TLRs/NF-κB pathway and the mRNA levels of inflammatory cytokines and chemokines were examined by Western blotting and PCR, respectively. Finally, the hepatic expression of pregnane X receptor (PXR) and farnesoid X receptor (FXR) and their target genes encoding metabolic enzymes and transporters was evaluated. GA significantly reversed liver necrosis and decreased plasma ALT and ALP activity. Plasma total bile acids, total bilirubin, and hepatic bile acids were also remarkably preserved. More importantly, the recruitment of inflammatory cells to hepatic sinusoids was alleviated. Additionally, the protein expression of TLR2, TLR4, and p-NF-κBp65 and the mRNA expression of CCL2, CXCL2, IL-1β, IL-6, and TNF-α were significantly decreased. Moreover, GA significantly increased the expression of hepatic FXR and its target genes, including BSEP, MRP3, and MRP4. In conclusion, GA protects against LCA-induced cholestatic liver injury by inhibiting the TLR2/NF-κB pathway and upregulating hepatic FXR expression.

## Introduction

Bile acids (BAs) are synthesized from cholesterol by hepatocytes and conjugated with either taurine or glycine. These primary BAs are then secreted into the duodenum from the bile duct and biotransformed by resident bacteria in the gut to secondary BAs such as lithocholic acid (LCA) and deoxycholic acid (DCA) (Russel, 2003). In the hepatocytes, phase I and phase II drug-metabolizing enzymes can catalyze the hydroxylation and sulfation or glucuronidation of BAs to increase their water solubility and reduce their toxicity. Moreover, BAs can also return to the liver in portal venous blood and are taken up into the liver by sodium taurocholate-cotransporting polypeptide (NTCP) and organic anion-transporting polypeptides (OATPs). Canalicular secretion is mediated by the bile salt export pump (BSEP). Multidrug resistance-associated proteins 3 and 4 (MRP3 and MRP4) and the organic solute transporter alpha-beta (OSTα/β), located in the basolateral membrane, are considered alternative routes when efficient export to the bile is compromised (Li and Chiang, 2013).

BA pool size and composition can be modulated by nuclear receptors in hepatocytes such as farnesoid X receptor (FXR) and pregnane X receptor (PXR). FXR positively regulates BSEP and OSTα/β expression and inhibits CYP7A and NTCP expression through an increase in small heterodimer partner (SHP) expression in the liver (Fiorucci et al., 2014). PXR target genes include phase I and phase II metabolism enzymes and transporters. Some of these enzymes and transporters are associated with BA metabolism and transport, such as cytochrome P450 3A11 (CYP3A11) and sulfotransferase family 2A member 1 (SULT2A1) (Wagner et al., 2005).

Cholestasis is a pathological state involving impaired bile synthesis, metabolism, and transport excretion that lead to the accumulation of BAs in the liver and damage to hepatocytes (Trauner et al., 1998). This pathological situation occurs in many diseases, such as biliary obstruction, biliary atresia, intrahepatic cholestasis, and cholangiocarcinoma. The accumulation of cytotoxic BAs can induce an inflammatory response and trigger hepatocyte apoptosis and necrosis. If left untreated, cholestasis will cause liver damage, liver fibrosis, cirrhosis, and even liver failure (Cai and Boyer, 2017). Currently, ursodeoxycholic acid (UDCA) and obeticholic acid (OCA) are the only two therapies for cholestasis approved by the US Food and Drug Administration (FDA). However, up to 40% of patients fail to respond to UDCA, and a cluster of adverse effects associated with the use of OCA has been reported (Zhang et al., 2018; Fiorucci et al., 2019). Therefore, there remains an unmet need for the treatment of cholestasis in the clinic.

Cholestatic liver injury was previously assumed to be caused by the detergent properties of an excessive accumulation of BAs (de Buy Wenniger and Beuers, 2010). As such, the therapeutic strategy for cholestasis has focused on limiting intracellular BA accumulation by increasing the efflux and metabolism of hepatic BAs (Zeng et al., 2017). BA homeostasis is mediated by transporters and metabolic enzymes, which are transcriptionally regulated by nuclear receptors (Zollner et al., 2010; Zhang et al., 2015; Floreani and Mangini, 2018). Furthermore, recent studies suggest that the cholestatic liver injury is related to the cytokine-mediated inflammatory response, and the hepatoprotective effects of anti-inflammatory approaches have been proven (Woolbright and Jaeschke, 2012; Cai and Boyer, 2017). The cholestatic inflammatory response is usually mediated by toll-like receptor (TLR) signaling. The synthesis and release of chemokines (e.g., CCL2 and CXCL2) increase upon the activation of TLRs, which can upregulate several transcription factors, including nuclear factor kappa-B (NF-κB). The chemokines recruit neutrophils and monocytes to damaged hepatocytes and aggravate the liver injury. Meanwhile, cholestatic liver damage has been associated with the marked release of inflammatory mediators, including IL-1β, IL-6, and TNF-α (Gujral et al., 2003; Allen et al., 2011; Li et al., 2017). For these reasons, the therapeutic strategy for cholestasis should reduce the cytokine-mediated inflammatory response via inhibition of the TLRs/NF-κB pathway as well as by maintaining BA homeostasis.

Glycyrrhizae, also known as licorice, is the most frequently used Chinese herb or food additive (Yu et al., 2018). We found that a glycyrrhizae decoction prepared from licorice root protected against ANIT-induced cholestatic liver injury of mice through inhibition of the TLRs/NF-κB signaling pathway (Su et al., 2021). Glycyrrhizin is the principal triterpene component of the glycyrrhizae decoction, and it is mainly metabolized to glycyrrhetinic acid (GA) through the intestinal flora (Akao et al., 1994; Su et al., 2021). Due to high exposure in the liver and a good therapeutic effect, GA can be a potent agent for cholestasis treatment in the clinic. Wu et al. (2018) reported that GA protected against ANIT-induced cholestasis, possibly via activation of FXR-mediated efflux transporters, with consequent attenuated dysregulation of BA homeostasis in the liver. Yan et al. (2018) determined that glycyrrhizin alleviates nonalcoholic steatohepatitis (NASH) by activating FXR and reducing inflammation responses, and the hepatoprotective effect was possibly attributed to GA. However, it remains unknown whether the hepatoprotective effect of GA on cholestatic liver injury results from upregulation of FXR-mediated efflux transporter and inhibition of the TLRs/NF-κB pathway.

LCA is hepatotoxic and has been widely used to produce a model of cholestatic liver damage (Fickert et al., 2006). Administration of LCA to mice leads to hepatic parenchyma damage and disruption of bile flow, which are similar to cholestatic liver disease. In addition to the focal areas of necrosis, an extensive accumulation of neutrophils and monocytes is also observed in this model (Delzenne et al., 1992). The aim of the present work was to evaluate the hepatoprotective effect of GA in LCA-induced cholestatic liver damage and to explore whether the effect results from both maintained BA homeostasis and an anti-inflammatory effect.

## Materials and Methods

### Animal Experiments

Male C57BL/6 mice (20 ± 2 g; 4 weeks old) were housed at the Department of Laboratory Animal Science, Shanghai University of Traditional Chinese Medicine in Shanghai, China. All animals were maintained on a 12-h light/dark cycle at a constant temperature (25 ± 2°C) and humidity (60%–70%). Mice were provided a standard diet and tap water *ad libitum* and allowed to acclimatize to the environment for 1 week prior to the experiment. The mice were randomly divided into four groups (*n* = 5 each): a vehicle group, an LCA group, and LCA groups treated with either 50 mg/kg GA (J0622A, Meilunbio, Dalian, China) or 45 mg/kg pregnenolone 16α-carbonitrile (PCN; C3884, APExBIO, Houston, TX, United States). The scheme of animal experiments was adapted from that of previous studies (Zhang et al., 2015; Kong et al., 2018; Fan et al., 2019). The GA dose used here (50 mg/kg) was chosen based on the literature (Sun et al., 2017; Wang et al., 2017; Wu et al., 2021) and our previous studies. Furthermore, GA at a dose of 50 mg/kg effectively protected against cholestatic liver injury (Wang et al., 2017; Yan et al., 2021). The GA and PCN were dissolved in DMSO and further diluted in corn oil with 2% DMSO in the final solution. The drugs were intraperitoneally injected once daily for 7 days. LCA (L6250, Sigma-Aldrich, St. Louis, MO, United States) was dissolved in corn oil and intraperitoneally injected (0.125 mg/g) twice daily from the fourth day (Supplementary Figure S1). Mice in the vehicle group received an equal volume (10 ml/kg) of 2% DMSO/corn oil. Mice were sacrificed 12 h after the final LCA injection. Blood and liver tissue samples were collected and stored at −80°C in a refrigerator for later testing. All protocols were approved by the guidelines of the Institutional Animal Ethics Committee at Shanghai University of Traditional Chinese Medicine (approval no. PZSHUTCM190510001).

### Biochemical Assay

The concentrations of alanine aminotransferase (ALT), alkaline phosphatase (ALP), aspartate aminotransferase (AST), total bilirubin (TBIL), and total BA (TBA) were tested in mouse plasma using commercially available kits and a biochemical analysis system (AU5800, Beckman Coulter, Germany).

### Histological Examination

Liver samples were fixed in 4% paraformaldehyde, embedded in paraffin, and cut into 5-μm slices. The sections were subsequently stained with hematoxylin and eosin (H&E) for the evaluation of necrosis. Slides were examined under an Olympus BX41 microscope at magnifications of 40× and 400×. The necrotic score, indicating the number of necrotic foci per low-power magnification field (40×) per slide, was determined according to the method of Rudich et al. (2009).

### Western Blot Analysis

Mouse liver tissues were extracted using ice-cold RIPA lysis buffer, and the protein concentrations were determined by a BCA protein assay kit. Equal amounts of protein extracts were separated by 8% sodium dodecyl sulfate–polyacrylamide gel electrophoresis (SDS-PAGE) and then transferred onto polyvinylidene difluoride (PVDF) membranes, blocked with 5% skimmed milk for 1 h at room temperature and incubated at 4°C overnight with the primary antibodies. Then, the membranes were incubated with horseradish peroxidase-conjugated secondary antibodies at room temperature for 1 h. Finally, the proteins were detected using an enhanced chemiluminescence (ECL) method and imaged using a ChemiDoc XRS system. The densities of the bands were assessed and normalized to the β-actin or GAPDH signals. The primary antibodies were as follows: TLR2 (1:1,000; sc-166900, Santa Cruz Biotechnology, Santa Cruz, CA, United States); TLR4 (1:1,000; sc-293072, Santa Cruz Biotechnology); p-NF-κBp65 (1:1,000; sc-166748, Santa Cruz Biotechnology); β-actin (1:1,000; sc-8432, Santa Cruz Biotechnology); FXR (1:500; 25055-1-AP, Proteintech, Chicago, IL, United States); GAPDH (1:10,000; 60004-1-lg, Proteintech); PXR (1:1,000; bs-2334R, Bioss, Beijing, China); NTCP (1:500; bs-1958P, Bioss); OATP1A1 (1:300; bs-0607R, Bioss); OST-β (1:300; bs-2128R, Bioss); MRP2 (1:500; bs-1092R, Bioss); MRP3 (1:1,000; 39909, Cell Signaling Technology, Danvers, MA, United States); MRP4 (1:1,000; 12857S, Cell Signaling Technology); and BSEP (1:1,000; DF9278, Affinity Biosciences). The secondary antibodies were as follows: goat anti-rabbit (1:10,000; MR-R100, Biotech, Shanghai, China) and goat anti-mouse (1:10,000; MR-G100, Biotech).

### Quantitative Real-Time PCR

Il-1β, Il-6, Tnf-α, Ccl2, and Cxcl2 mRNA levels were determined by quantitative real-time PCR (qRT-PCR). Total RNA was isolated from mouse liver tissues using TRIzol, cDNA was synthesized, and RT-qPCR was performed with primers and a 2 × SYBR Green qPCR Master Mix (11203ES03, Yeasen, Shanghai, China) in a StepOne Plus PCR System (Thermo Fisher Scientific, Waltham, MA, United States). Mouse *Gapdh* was run for each sample to normalize expression levels. The relative amount of mRNA in each sample was determined using the ΔΔCT method. The sequences of the primers used are listed in Table 1. The amplification reactions were performed as follows: 95°C for 5 min, followed by 40 cycles of 95°C for 10 s, and 60°C for 30 s. The melting curve reactions were set as follows: 95°C for 15 s, 60°C for 1 min with an increment of 0.15°C/s to 95°C, and then a 15-s hold.

**TABLE 1:** Primer sequences for RT-PCR.

Gene	RefSeq	Product length (bp)	Forward primer (5′–3′)	Reverse primer (5′–3′)
*Cyp3a11*	NM_007818.3	72	GGA​TGA​GAT​CGA​TGA​GGC​TCT​G	CAG​GTA​TTC​CAT​CTC​CAT​CAC​AGT
*Ugt1a1*	NM_201645.2	118	GCT​TCT​TCC​GTA​CCT​TCT​GTT​G	GCT​GCT​GAA​TAA​CTC​CAA​GCA​T
*Sult 2a1*	NM_001111296.2	157	GGA​AGG​ACC​ACG​ACT​CAT​AAC	GAT​TCT​TCA​CAA​GGT​TTG​TGT​TAC​C
*Cyp7a1*	NM_007824.3	255	GAA​CCT​CCT​TTG​GAC​AAC​GGG	GGA​GTT​TGT​GAT​GAA​GTG​GAC​AT
*IL-1β*	NM_008361.4	54	TGA​CGG​ACC​CCA​AAA​GAT​G	TGG​ACA​GCC​CAG​GTC​AAA​G
*IL-6*	NM_031168.2	140	ACT​TCC​ATC​CAG​TTG​CCT​TCT​TGG	TTA​AGC​CTC​CGA​CTT​GTG​AAG​TGG
*Tnf-α*	NM_013693.3	138	GGT​GCC​TAT​GTC​TCA​GCC​TCT​T	GCC​ATA​GAA​CTG​ATG​AGA​GGG​AG
*Ccl2*	NM_011333.3	100	CCA​GCA​AGA​TGA​TCC​CAA​TGA	TCT​CTT​GAG​CTT​GGT​GAC​AAA​AAC
*Cxcl2*	NM_009140.2	107	CCA​ACC​ACC​AGG​CTA​CAG​G	GCG​TCA​CAC​TCA​AGC​TCT​G
*Gapdh*	NM_008084.3	158	TGT​GAA​CGG​ATT​TGG​CCG​TA	ACT​GTG​CCG​TTG​AAT​TTG​CC

### Quantitation of Bile Acids by LC-MS/MS

BA extraction from the liver was performed according to a previously described method (Zhang et al., 2018). A mass of 100 mg of liver tissue was homogenized and vortex-mixed with 900 μl 70% acetonitrile. Then, the mixture was centrifuged at 10,000 rpm for 10 min, the supernatant was collected, and 100 nM d5-LCA was added as an internal standard for analysis. BAs were detected by a Shimadzu LC-20AD liquid chromatography (Shimadzu, Kyoto, Japan) coupled to a 4000 Q-Trap mass spectrometer (Applied Biosystems Sciex, ON, Canada). Chromatographic separation was achieved using an Eclipse XDB-C18 column (4.6 mm × 150 mm, 5 μm) at room temperature. The gradient of the mobile phase comprising solvent A (4 mM acetate ammonium and 0.05% formic acid) and solvent B (4 mM acetate ammonium and 0.05% formic acid in acetonitrile/methanol [v/v = 3/1]) with a flow rate of 1 ml/min is as follows: 0–2 min, 70% A; 2–6 min, 70%–67% A; 6–12 min, 67%–55% A; 12–19 min, 55%–10% A; 19–23 min, 10% A; and 23.1–29 min, 70% A. Negative ionization mode and multiple reaction monitoring (MRM) mode were selected for quantification. The precursor/product ion mass transitions monitored were as follows: *m*/*z* 375.1→375.1 for LCA, *m*/*z* 482.3→79.8 for TLCA, *m*/*z* 373.1→373.1 for 3keto-LCA, *m*/*z* 407.1→407.1 for CA/α-MCA/β-MCA/ω-MCA, *m*/*z* 514.3→79.7 for TCA/T-α-MCA/T-β-MCA, *m*/*z* 391.3→391.3 for CDCA/DCA/HDCA/UDCA/MDCA, *m*/*z* 498.2→79.8 for TCDCA/TDCA/TCDCA/TUDCA, and *m*/*z* 380.3→380.3 for d5-LCA. The ion spray voltage was set at −3,500 V, and the ion source temperature was set at 500°C. Nitrogen was used as the collision gas with an intensity set as a medium. Curtain gas, ion source gas 1, and ion source gas 2 were set at 25, 60, and 60 psi, respectively. Data acquisition and analysis were conducted with Analyst software (version 1.5.2, Applied Biosystems, CA).

### Statistical Analysis

Data were analyzed using SPSS 21.0 software and are expressed as mean ± standard deviation. Differences between groups were calculated by one-way analysis of variance (ANOVA) and Dunnett’s test. Differences were considered statistically significant at *p* < 0.05.

## Results

### Glycyrrhetinic Acid Protects Against Lithocholic Acid-Induced Cholestatic Liver Injury in Mice

**FIGURE 1:**
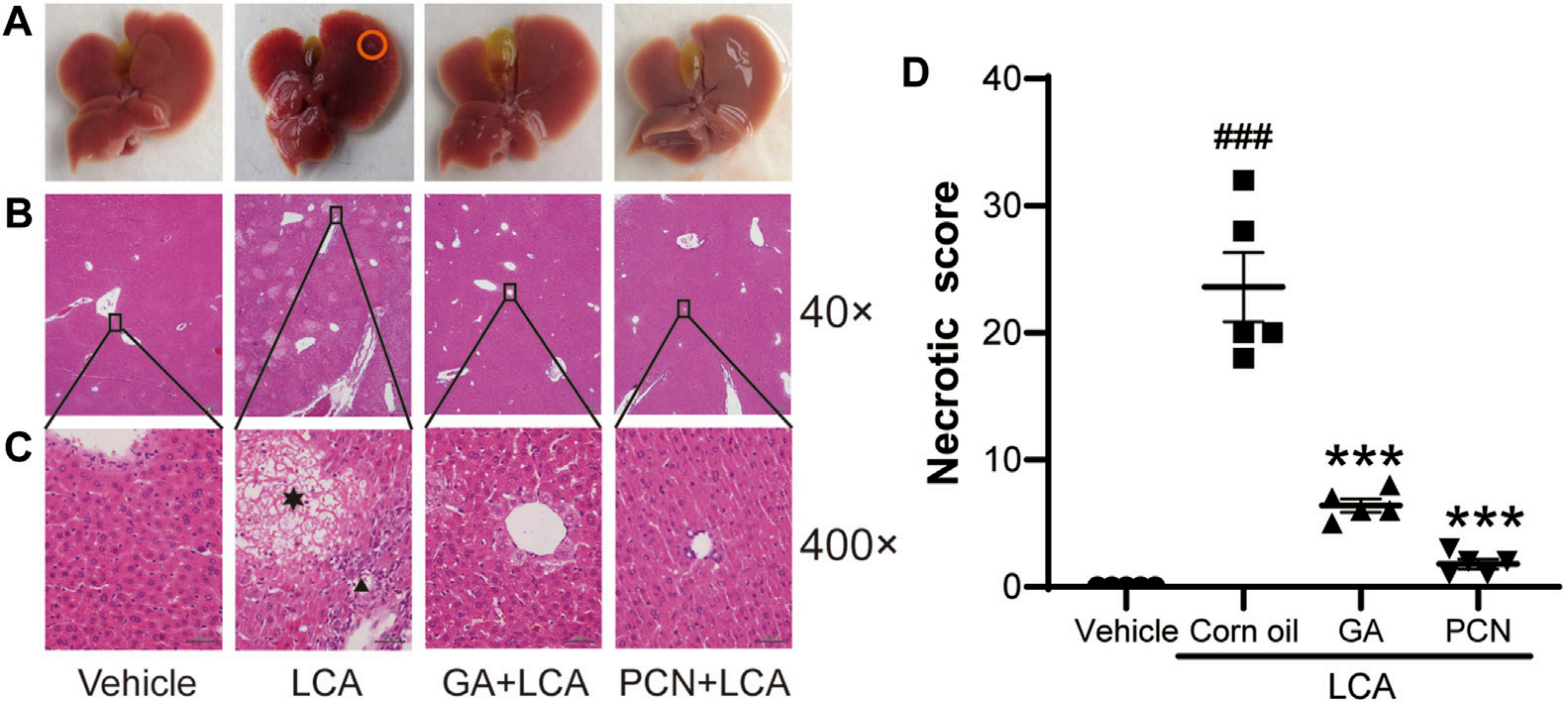
Effects of GA on histopathological changes of hepatic tissues. **(A)** Gross inspection, bile infarcts are marked by red circles; **(B)** low magnification (40×); **(C)** high magnification (400×). Hepatocyte necrosis is marked by asterisks, and portal inflammation cells are marked by arrowheads. **(D)** Necrotic score of H&E-stained liver sections (low magnification of 40×). Data are shown as mean ± SD (*n* = 5). #*p* < 0.05 vs. vehicle group; ^∗∗∗^ *p* < 0.001 vs. LCA group.

**FIGURE 2:**
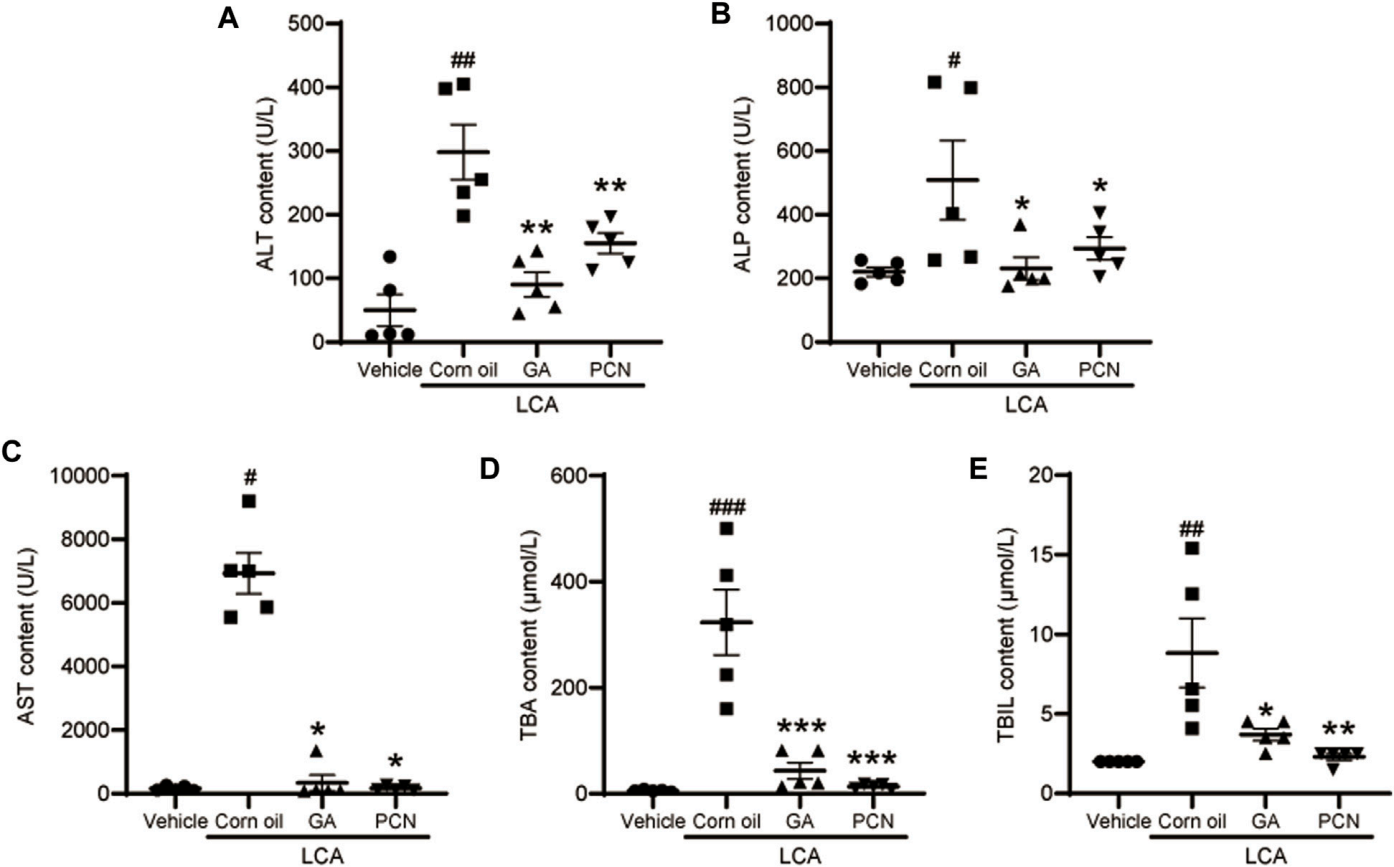
Effect of GA on plasma levels of biochemical indicators. **(A)** ALT; **(B)** ALP; **(C)** AST; **(D)** TBA; and **(E)** TBIL. Data are shown as mean ± SD (*n* = 5). #*p* < 0.05, ##*p* < 0.01, and ###*p* < 0.001 vs. vehicle group; ^∗^ *p* < 0.05, ^∗∗^ *p* < 0.01, and ^∗∗∗^ *p* < 0.001 vs. LCA group.

Upon gross inspection, the gallbladder volume was increased in the LCA group compared with the vehicle group. To investigate the protective effect of GA on LCA-induced cholestatic liver injury in mice, liver slices were stained with H&E. Multiple necrotic foci and inflammatory cell infiltration to the hepatic sinus were observed in the LCA group, but the histological pattern was significantly attenuated by co-treatment with GA or PCN (Figure 1). The pathological improvement was confirmed using biochemical indicators (Figure 2). Compared with the vehicle group, the plasma TBA, TBIL, ALT, ALP, and AST levels were significantly higher in the LCA group, reaching 59.6-, 3.4-, 5.0-, 1.3-, and 40.7-fold higher than those in the vehicle group, respectively. The increase was markedly reversed by co-treatment with GA or PCN. Compared with the LCA group, GA decreased plasma TBA, TBIL, ALT, ALP, and AST levels by 89.5%, 60.4%, 69.8%, 55.1%, and 95.1%, respectively, while PCN reduced plasma TBA, TBIL, ALT, ALP, and AST levels by 95.9%, 74.0%, 32.6%, 42.2%, and 97.5%, respectively. Thus, the biochemical and pathology results showed that GA and PCN had protective effects in LCA-induced liver injury in mice.

### Glycyrrhetinic Acid Reduces the Bile Acid Accumulation Induced by Lithocholic Acid in Mouse Liver

**FIGURE 3:**
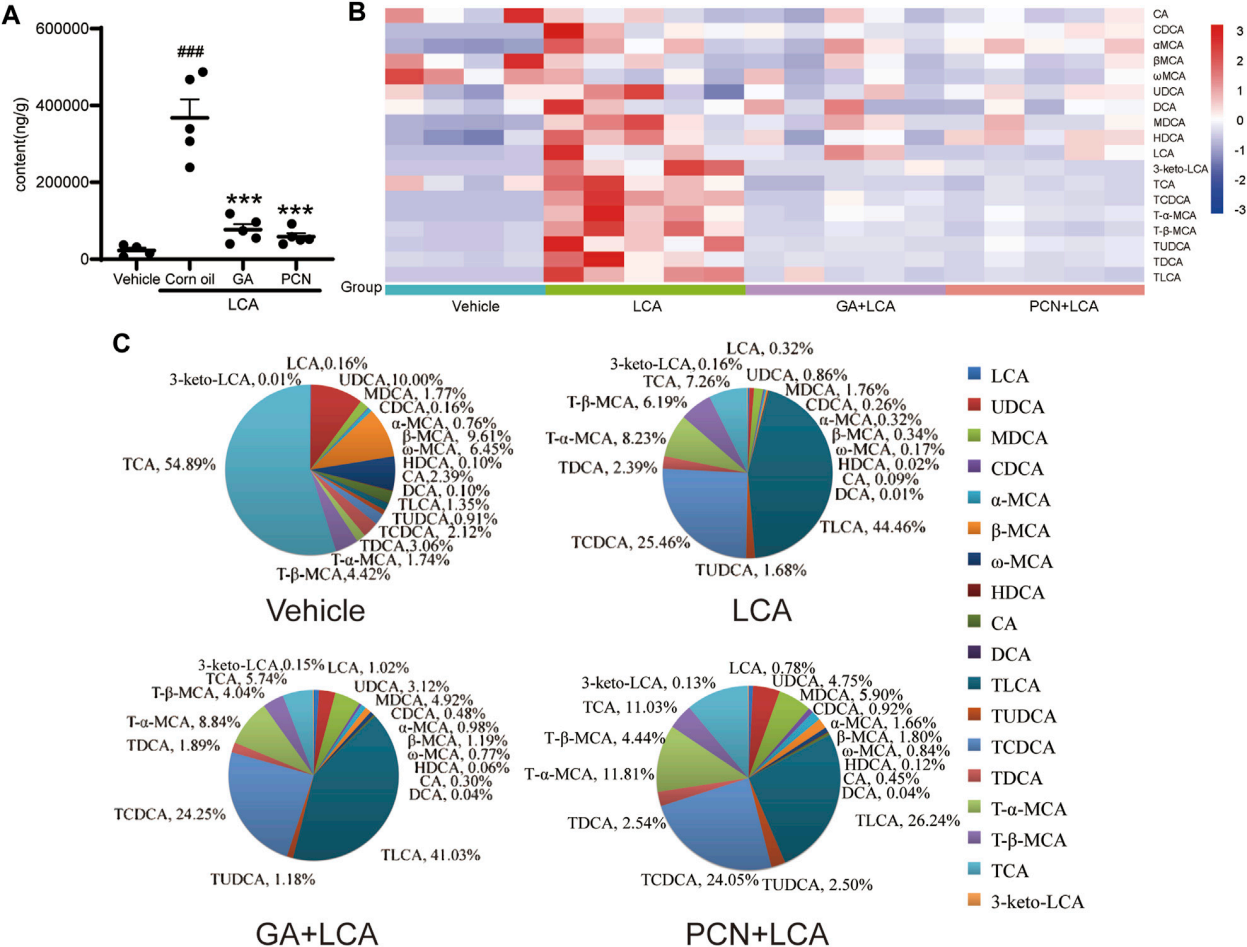
Effect of GA on the BA composition in the liver from LCA-induced model mice. **(A)** Amount of total detected BAs; **(B)** heatmap of hepatic individual BAs (blue represents downregulating, and red represents upregulating, compared with the vehicle group); **(C)** bile acid composition of the hepatic BA pool. Data are expressed as mean ± SD (*n* = 5). ###*p* < 0.001 vs. vehicle group; ^∗∗∗^ *p* < 0.001 vs. LCA group.

To compare the changes in the hepatic BAs of the mice in each group, 18 individual BAs in mouse liver were quantified by LC-MS/MS. The total amount of detected BAs (total BA) in the liver was significantly increased in the LCA group, reaching 13.33-fold higher than that in the vehicle group. In contrast, GA and PCN significantly reduced the amount of total BA, which decreased to 76.0% and 84.2% of that in the LCA group, respectively (Figure 3A). To clarify the changes in the individual hepatic BAs, a heatmap of BAs was created (Figure 3B). The levels of both unconjugated and tauro-conjugated BAs in the liver were significantly increased in the LCA group compared with the vehicle group. Treatment with GA and PCN reduced the BAs to near-normal levels.

To further compare the differences in the BA profile among each group, we evaluated the proportion of individual BAs in each group (Figure 3C). TCA (54.89%), UDCA (10.00%), and β-MCA (9.61%) were the major BAs in the vehicle group. However, the hepatic BA profile was completely altered in the LCA group, with TLCA and TCDCA substantially increased to 44.46% and 25.46%, respectively, and TCA decreased to 7.26%. Compared with the LCA group, the BA pool size was sharply decreased in the GA group, but the percentage of individual tauro-conjugated BAs, including TLCA, TCDCA, T-β-MCA, TUDCA, TDCA, and TCA, was similar due to BSEP-dependent efflux into the bile duct. For unconjugated BAs independent of BSEP-mediated efflux, the proportions showed a tendency to increase. Interestingly, although PCN markedly decreased the BA pool size, individual tauro-conjugated BAs decreased at different proportions. Among the seven tauro-conjugated BAs identified in the liver, only the TLCA proportion decreased from 44.46% to 26.24% compared with the LCA group. The proportions of TCDCA and TDCA were similar in the two groups, and TCA, T-α-MCA, and TUDCA in the PCN groups showed a tendency to increase compared with the LCA group. In contrast, the percentages of unconjugated BAs were markedly increased in the PCN group.

### Glycyrrhetinic Acid Inhibits the TLRs/NF-κB Signaling Pathway and Reduces the Release of Cytokines

**FIGURE 4:**
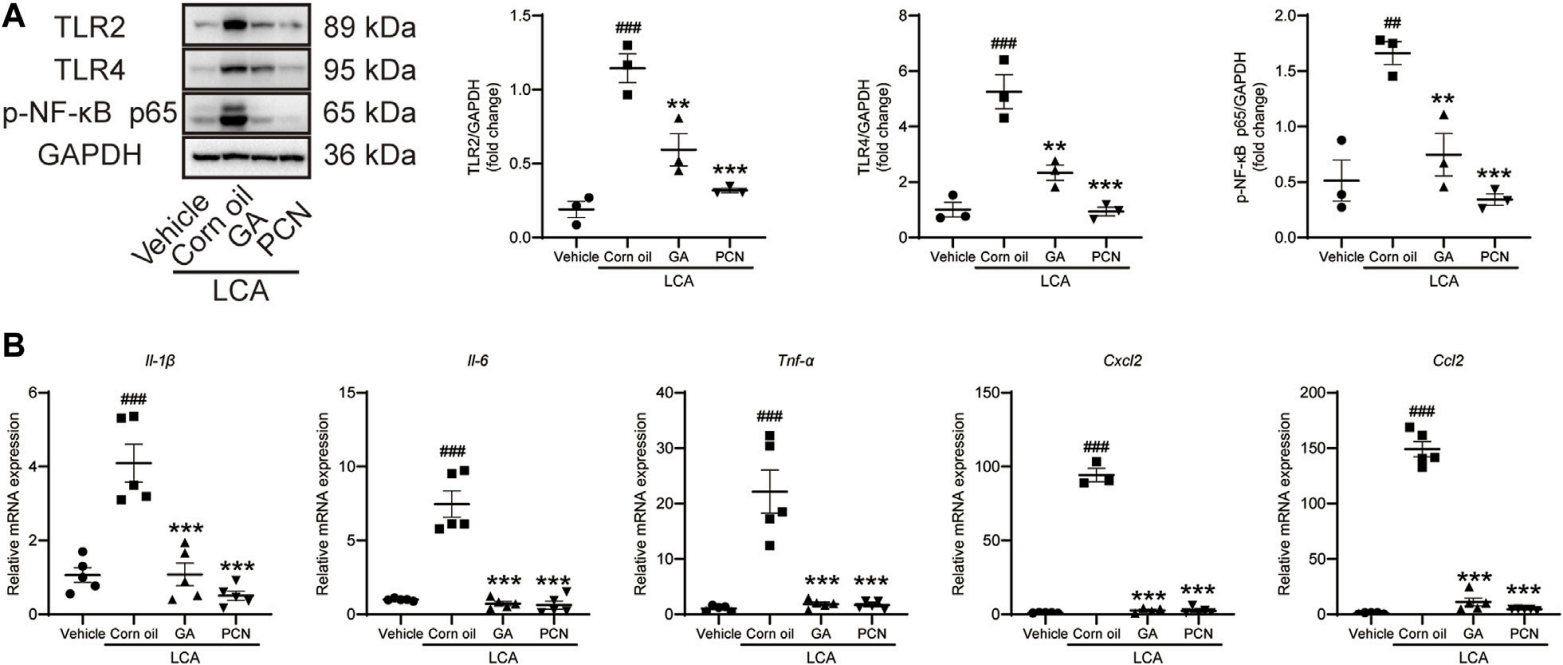
GA suppressed hepatic TLR_S_/NF-κB signal pathway in mice induced by LCA. **(A)** Proteins of liver extracts were evaluated by Western blotting for the determination of TLR2, TLR4, and p-NF-κB-p65 levels. **(B)** mRNA levels of *Il-1β*, *Il-6*, *Tnf-α*, *Ccl2*, and *Cxcl2* in the hepatic tissues were measured by RT-qPCR. Data are shown as mean ± SD (*n* = 5). ##*p* < 0.01 and ##*p* < 0.001 vs. vehicle group; **p < 0.01 and ***p < 0.001 vs. LCA group.

To investigate whether GA could ameliorate BA-triggered inflammatory responses via the TLR/NF-κB signaling pathway, the hepatic protein expression of TLR2, TLR4, and p-NF-κB p65 and the mRNA levels of cytokines and chemokines were determined by Western blotting and RT-PCR, respectively. The levels of TLR2, TLR4, and p-NF-κB p65 were increased in the LCA group compared with the vehicle group, and the elevation was significantly reversed by GA intervention. Additionally, co-treatment with PCN also resulted in restoration of TLR2, TLR4, and p-NF-κB expression (Figure 4A). LCA-treated mice showed significant increases in the expression of *Il-1β*, *Il-6*, *Tnf-α*, *Ccl2*, and *Cxcl2* mRNAs compared with the vehicle group. GA and PCN significantly inhibited the overproduction of these cytokines and chemokines (Figure 4B).

### Glycyrrhetinic Acid Affects Farnesoid X Receptor and Downstream Drug‐Metabolizing Enzymes and Transporters Involved in Bile Acid Homeostasis

**FIGURE 5:**
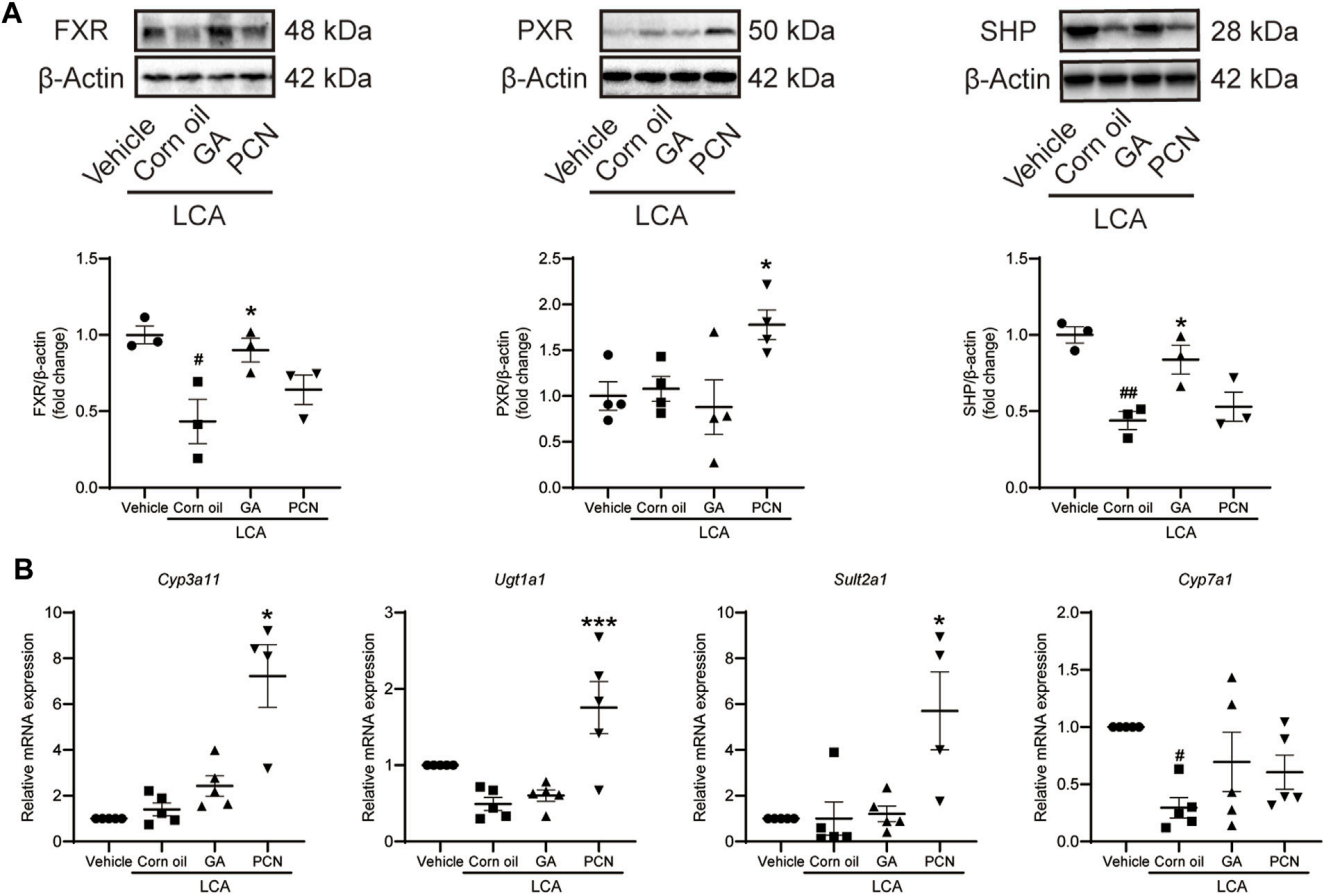
GA regulated hepatic expression of FXR, PXR, and metabolic enzymes involved in BA detoxification in LCA-induced cholestatic liver injury. **(A)** Protein levels of FXR, PXR, and SHP were detected by Western blot analysis; **(B)** mRNA levels of *Cyp3a11*, *Ugt1a1*, *Sult2a1*, and *Cyp7a1* were determined by RT-qPCR. Data are presented as mean ± SD (*n* = 5). #*p* < 0.05 and ##*p* < 0.01 vs. vehicle group; ^∗^ *p* < 0.05 and ^∗∗∗^ *p* < 0.001 vs. LCA group.

To elucidate the choleretic effects of GA on LCA-induced cholestasis, we measured the protein expression of nuclear receptors, including FXR and PXR. In the LCA group, LCA significantly decreased the baseline FXR protein expression but showed little effect on PXR protein expression. The expression level of FXR, but not that of PXR, was significantly upregulated in the GA group compared with the vehicle group. In contrast, PCN significantly increased PXR protein levels but had little effect on FXR (Figure 5A). Similarly, the protein expression of SHP was downregulated by LCA, which was reversed by GA, but not by PCN. The mRNA expression of BA-metabolizing enzymes, including *Cyp3a11*, *Ugt1a1*, *Sult2a1*, and *Cyp7a1*, was further evaluated to validate the GA activation of nuclear receptors (Figure 5B). No significant difference was observed in the expression of *Cyp3a11*, *Ugt1a1*, or *Sult2a1* mRNAs between the vehicle and LCA groups, but hepatic *Cyp7a1* was significantly decreased by LCA administration. PCN treatment enhanced the mRNA expression of *Cyp3a11*, *Ugt1a1*, and *Sult2a1* compared with the LCA group. In contrast, the expression levels of these BA-metabolizing enzymes were not significantly altered in the GA group compared with the LCA group. Neither PCN nor GA significantly increased the mRNA expression of *Cyp7a1*.

**FIGURE 6:**
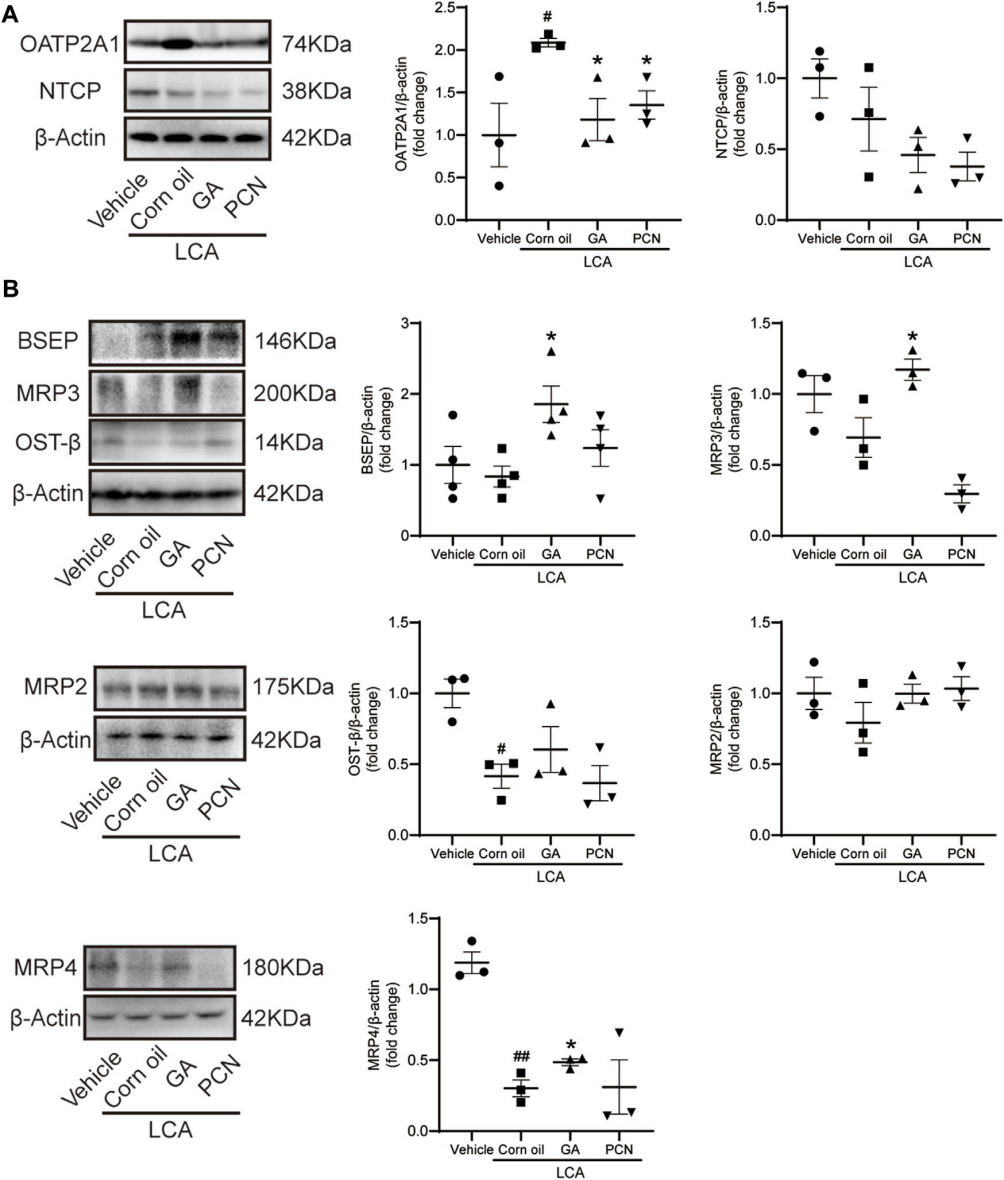
GA altered hepatic expression of bile acid transporters in LCA-induced cholestatic liver damage. **(A)** Western blot was used to measure the expression of uptake transporters, including NTCP and OATP2A1; **(B)** protein levels of efflux transporters, including BSEP, MRP2, MRP3, MRP4, and OST-β. Data are presented as mean ± SD (*n* = 5). #*p* < 0.05 and ##*p* < 0.01 vs. vehicle group; ^∗^ *p* < 0.05 vs. LCA group.

We measured the protein levels of transporters involved in BA transport, including NTCP, OATP2A1, BSEP, MRP2, MRP3, MRP4, and OST-β. Compared with the vehicle group, LCA administration resulted in a significant increase in the BA uptake transporter OATP2A1 and a decrease in the BA efflux transporters MRP4 and OST-β (Figure 6A), which were consistent with the pronounced elevation of BAs in the liver. Compared with the LCA group, GA dramatically increased the protein levels of the canalicular BA efflux transporter BSEP and basolateral BA efflux transporters MRP3 and MRP4 but reduced the expression of the BA uptake transporter OATP2A1 (Figure 6B). However, PCN had little effect on the protein expression of BSEP, MRP2, MRP3, MRP4, and OST-β. This finding suggested that the protective effect of GA against cholestasis may be a result of enhanced BA output, which is mediated by FXR activation.

## Discussion

The present study demonstrated the hepatoprotective effects and mechanisms of GA in LCA-induced cholestatic liver injury. LCA is a hydrophobic secondary BA formed in the intestine by bacterial 7α-dehydroxylation from chenodeoxycholic acid (CDCA). Under normal conditions, LCA is mainly transformed into tauro-conjugates in the liver and transported into the bile duct. However, an excessive amount of LCA can cause a loss of gap junction proteins and a collapse of the bile osmotic gradient, which results in decreased movement of BAs across membranes and their subsequent accumulation in the liver (Trauner et al., 1998). Administration of the hydrophobic BA LCA results in severe liver damage, manifested as bile infarcts, segmental bile duct obstruction, and destructive cholangitis, which are analogous to the characteristics and histology of primary sclerosing cholangitis, a prototypic cholestatic liver disease (Fickert et al., 2006). Thus, LCA has been commonly used to prepare a cholestasis model in experiment animals (Zeng et al., 2016; Kong et al., 2018; Fan et al., 2019).

PCN has been reported in many studies to significantly ameliorate LCA-induced cholestasis and hepatotoxicity (Chai et al., 2013; Zhang et al., 2015; Zeng et al., 2016). PCN, a potent agonist of PXR in rodents, can induce *Cyp3a* and *Sult2a* to catalyze LCA hydroxylation and sulfation, which not only increase the solubility and excretion of LCA but also reduce its toxicity (Kliewer et al., 1998). The present findings showed that LCA induced marked hepatic damage in the form of infiltration of inflammatory cells around the bile duct and severe liver necrosis. Moreover, gallbladder volume was increased due to LCA activation of the G protein-coupled BA receptor (Gpbar1), also known as TGR5 (Pols et al., 2011). The plasma TBA level in the LCA group was 60.6-fold higher than that of the vehicle group, indicating marked hepatic BA accumulation in the liver of mice. The levels of ALT and ALP were clearly higher than those in the vehicle group, suggesting cholestatic liver damage. Concomitant GA or PCN use resulted in restoration of the blood biochemical indices and significantly improved liver pathology, with reduced necrosis and infiltration of inflammatory cells. These results suggest that GA has a protective effect on LCA-induced liver injury comparable to that of PCN.

Excessive intrahepatic accumulation of BAs, including LCA, has been associated with cholestatic liver damage (Beilke et al., 2008). Thus, reducing hepatic BA accumulation by enhancing metabolism and transportation is one of the therapeutic strategies for cholestasis. After LCA administration, LCA and other BAs are taken up from sinusoidal blood into hepatocytes by OATP2A1 and NTCP, located on the basolateral membrane. The BAs are then immediately conjugated with taurine by BA CoA: amino acid N-acyltransferase (BAAT), hydroxylated by CYP3A11, glucuronidated by UGT1A1, or sulfated by SULT2A1 (Chai et al., 2013). The canalicular efflux transporters BSEP and MRP2 are highly expressed in hepatocytes and contribute to the excretion of BAs from hepatocytes into the bile duct. The transporters MRP3, MRP4, and OST-β, which are expressed on the basolateral membrane, mainly mediate the transport of BAs into the blood as an alternative pathway (de Buy Wenniger and Beuers, 2010).

LCA can be hydroxylated to form murideoxycholic acid (MDCA), 3-ketocholanoic acid (3KCA), hyodeoxycholic acid (HDCA), and CDCA by 6β-, 3β-, 6α-, and 7α-hydroxylation, respectively (Deo and Bandiera, 2009; Zhang and Klaassen, 2010). Consistently, our data indicated that LCA administration significantly increased the levels of MDCA, 3KCA, HDCA, and CDCA in the mouse liver. Nonetheless, many of the polyhydroxy metabolites produced by LCA in the liver were not detected due to the lack of a reference substance. CYP3A has been suggested to mediate LCA hydroxylation in the mouse. Zhang et al. (2012) reported that hydroxylation is a pathway for detoxifying high concentrations of monohydroxy BAs, such as TLCA and TMCA, in the livers of bile duct ligation mice. Meanwhile, Schölmerich et al. (1984) reported that hepatotoxicity is inversely proportional to the degree of hydroxylation. Glucuronidation and sulfation are also believed to be important for detoxifying BAs in the liver. LCA and TLCA could be transformed into 3-sulfate and 3-glucuronide conjugates, and both make BAs more hydrophilic and facilitate their elimination in the feces and urine. Importantly, the hepatic formation of 3α-sulfated LCA and TLCA mediated by SULT2A1 is the rate-limiting step for the efficient detoxification of both BAs via renal and fecal excretion, thereby reducing hepatic BA accumulation (Kitada et al., 2003; Fickert et al., 2006; Miyata et al., 2006). PCN activates PXR to induce the expression of its downstream drug-metabolizing enzymes, such as CYP3A11, UGT1A1, and SULT2A1 (Staudinger et al., 2001; Beilke et al., 2008). Interestingly, in our study, liver *Cyp3a11* and *Sult2a1* mRNA levels were markedly elevated in the PCN group. Consequently, the total liver BA pool and the proportion of TLCA were both significantly decreased. The results indicated that PCN induced *Cyp3a11* and *Sult2a1* to accelerate the detoxification and secretion of BAs. However, the induction efficacy of GA for the mRNA expression of *Cyp3a11*, *Ugt1a1*, and *Sult2a1* was clearly inferior to that of PCN. FXR plays important regulatory roles in repressing BA synthetic enzymes, inhibiting hepatic uptake transporter, and inducing bile efflux transporters in the liver (Stedman et al., 2006). Pretreatment with GA sharply decreased the BA pool size, but the percentage of individual tauro-conjugated BAs was similar because the BSEP efflux of these substrates into the bile duct was at the same proportion. In addition, GA also induced the expression of efflux transporters (MRP3 and MRP4) and reduced the expression of uptake transporter (OATP2A1). Thus, GA confers hepatoprotection against LCA-induced cholestasis by upregulating FXR-associated transporters, thereby reducing the BA burden in hepatocytes. BA hydrophobicity is a determinant of toxicity and protection, with the more hydrophobic BAs causing greater levels of hepatocyte injury (Penman et al., 2019). Retention of hydrophobic BAs may cause cell membrane disruption due to detergent properties, which contribute to hepatic inflammatory responses and, finally, cell necrosis (Perez and Briz, 2009; Cai and Boyer, 2017). We found that treatment with GA and PCN decreased the amounts of hepatic hydrophobic BAs (LCA, DCA, and CDCA) and increased that of hydrophilic BA (UDCA), indicating their potential to reduce hepatocyte inflammatory responses and liver injury. Our data agree with previous results that GA may protect against ANIT-induced cholestatic liver injury by activating FXR and downstream transporters (Yan et al., 2021). Meanwhile, we found that GA exerted little effect on PXR activation, further supporting that FXR is the critical factor contributing to the choleretic effects of GA.

In addition to reducing hepatic BA accumulation in cholestasis, the therapeutic intervention should also include the targeting of cytokine-induced inflammatory liver injury (Woolbright and Jaeschke, 2019). Hepatocytes injured by LCA can release sterile mediators referred to as damage-associated molecular patterns (DAMPs), such as high mobility group box 1 (HMGB1) and mitochondrial DNA (mtDNA). The DAMPs can bind to TLRs expressed on Kupffer cells residing in the liver to further stimulate inflammatory responses, resulting in the establishment of a highly hepatotoxic feedforward cycle of inflammation and cell death (Dragomir et al., 2011; Tong et al., 2014; Shi et al., 2020). The expression levels of hepatic TLR2 and TLR4 were increased in the LCA group, accompanied by elevated levels of phosphorylated NF-κBp65 as well as the mRNA levels of cytokines and chemokines. The effects were attenuated after GA administration. Furthermore, GA had significant inhibition against LPS-induced TLR2 and TLR4 expressions in RAW264.7 cells, and the expression of chemokines was markedly diminished in *Tlr2* ^−/−^ cells (data not shown). These findings suggested that the hepatoprotective effects of GA on LCA-induced cholestatic liver injury might be mediated through inhibition of the TLR/NF-κB pathway. Similar findings have been reported by Yan et al. (2021), and our findings further indicate that TLRs may be the intervention target of GA. Nevertheless, our current work is limited in its investigation of the dose-dependent effect of GA in LCA mice, and further research is necessary to establish a clearer relationship between the dose and hepatoprotective effects of GA. Intriguingly, in addition to the induction of drug-metabolizing enzymes through PXR activation, PCN could also interact with NF-κB to downregulate the expression of chemokines, including CCL2 and CXCL2 (Okamura et al., 2020). Our data showed that PCN downregulated p-NF-kB expression and the subsequent mRNA expression of IL-6, IL-1β, TNF-α, CCL2, and CXCL2 in LCA-induced cholestatic mice. Meanwhile, decreased expression of TLR2 was observed in PCN-treated mice in this study. Murugan et al. (2019) reported that pregnenolone, the homolog of PCN, inhibited the secretion of TNF-α and IL-6 by suppressing the protein expression of TLR2. Further work is required to clarify whether PCN can downregulate the NF-κB-associated inflammatory pathway mediated by TLR2. Moreover, FXR can upregulate SHP in Kupffer cells to inhibit pro-inflammatory responses in the liver (Jin et al., 2020). In addition, FXR may limit NF-κB activation by stabilizing nuclear receptor corepressor 1 (Fiorucci et al., 2018). It would be interesting to investigate the anti-inflammatory effects mediated by nuclear receptors and to explore the intervention effects of GA and PCN in macrophages.

In summary, GA has a protective effect against LCA-induced cholestatic liver injury in mice, due to its ability to upregulate the FXR-associated pathway and prevent the activation of the TLR/NF-κB signaling pathway. PCN protects against LCA-induced liver damage by activating PXR and the downstream CYP3A11 and SULT2A1 and inhibiting the release of chemokines and cytokines by interfering with NF-κB in the liver of mice (Supplementary Figure S2). In addition to LCA-induced cholestasis, we would like to investigate the treatment and prevention effects of GA, separately, in a chronic cholestasis model. There is evidence to suggest that GA is effective in chronic liver disease models, including those of NASH and nonalcoholic fatty liver disease (Sun et al., 2017; Yan et al., 2018). Moreover, the protective effect of GA on NASH may be ascribed to the maintained BA homeostasis and to anti-inflammatory effects (Yan et al., 2018), which are also the main intervention approaches for cholestasis. Thus, we assume that GA may exert hepatoprotective effects in animal models of chronic cholestasis, which merits further research. This information could provide new insights into the hepatoprotective effects of GA on cholestasis through choleretic and anti-inflammatory mechanisms.

## Data Availability

The original contributions presented in the study are included in the article/Supplementary Material; further inquiries can be directed to the corresponding authors.
