# Supplementary figures and images for: Hepatoprotective Effects of Glycyrrhetinic Acid on Lithocholic Acid-Induced Cholestatic Liver Injury Through Choleretic and Anti-Inflammatory Mechanisms
Source: Front Pharmacol. 2022 May 31;13:881231. doi: 10.3389/fphar.2022.881231 (PMC9194553; doi:10.3389/fphar.2022.881231)

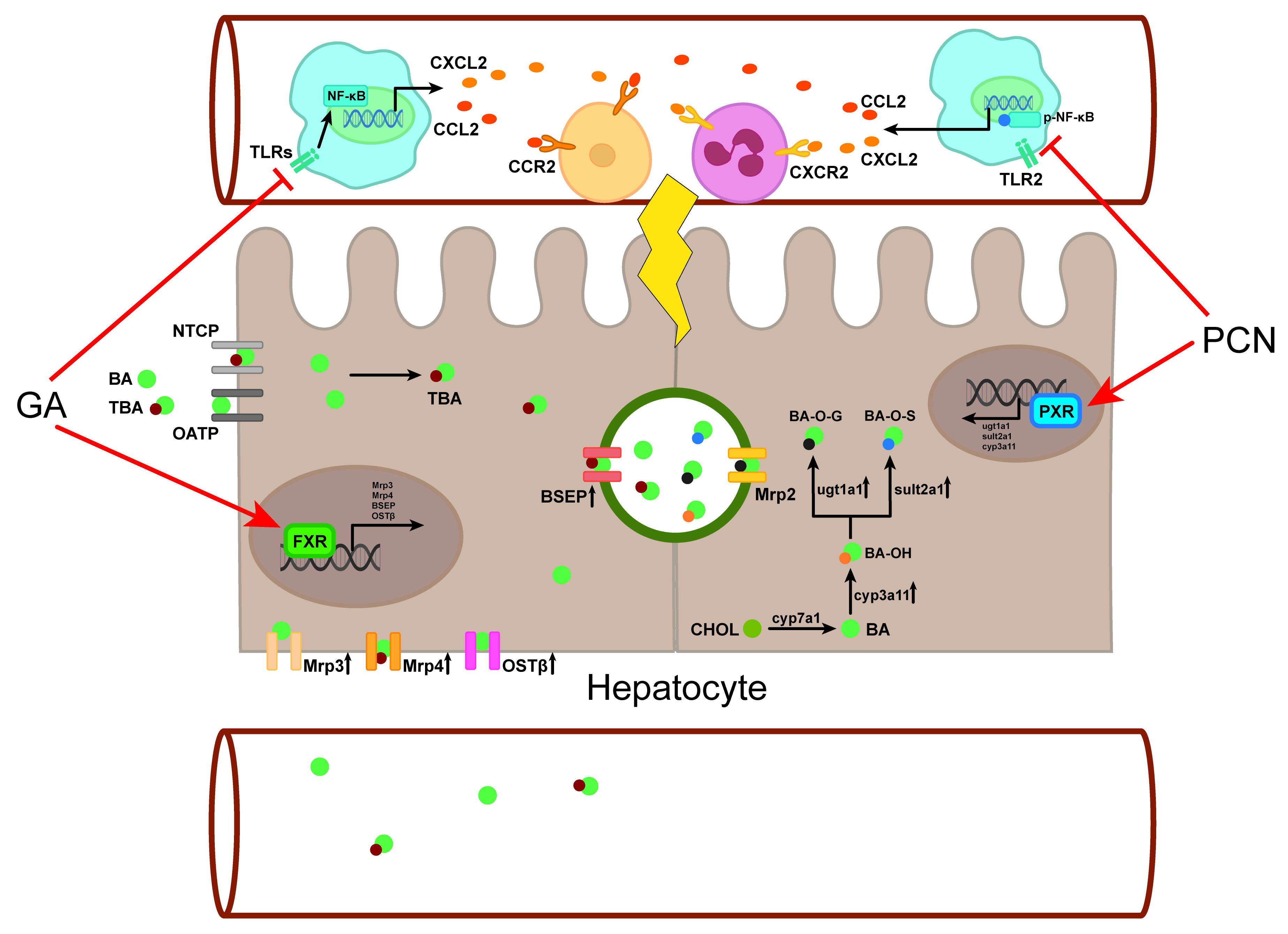

Supplement: Supplementary file 1 [file Image2.TIF]

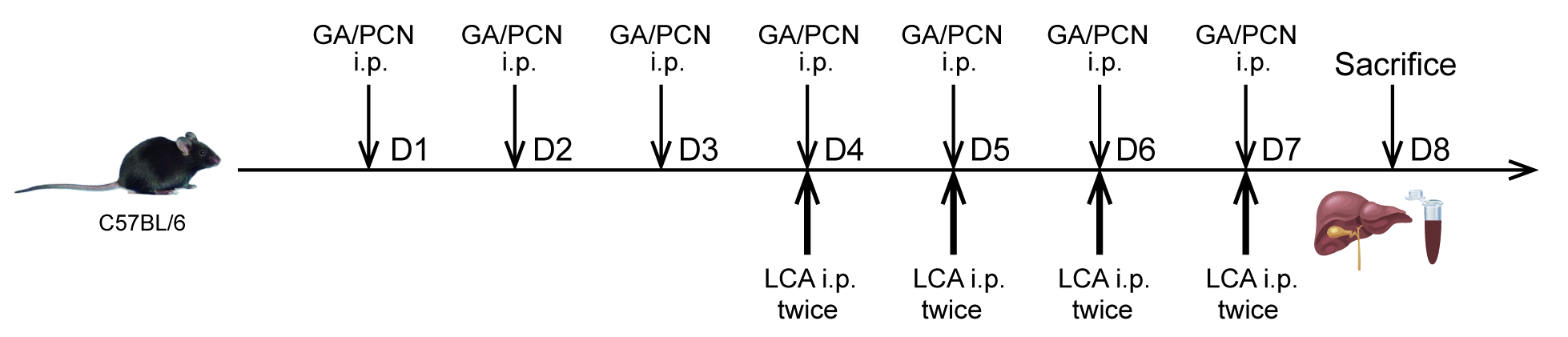

Supplement: Supplementary file 2 [file Image1.TIF]
